# Modeling and Mapping the Probability of Occurrence of Invasive Wild Pigs across the Contiguous United States

**DOI:** 10.1371/journal.pone.0133771

**Published:** 2015-08-12

**Authors:** Meredith L. McClure, Christopher L. Burdett, Matthew L. Farnsworth, Mark W. Lutman, David M. Theobald, Philip D. Riggs, Daniel A. Grear, Ryan S. Miller

**Affiliations:** 1 Conservation Science Partners, Truckee, California, United States of America; 2 Department of Biology, Colorado State University, Fort Collins, Colorado, United States of America; 3 Center for Epidemiology and Animal Health, Animal and Plant Health Inspection Service, United States Department of Agriculture, Fort Collins, Colorado, United States of America; 4 National Wildlife Disease Program, Animal and Plant Health Inspection Service, United States Department of Agriculture, Fort Collins, Colorado, United States of America; Università degli Studi di Napoli Federico II, ITALY

## Abstract

Wild pigs (*Sus scrofa*), also known as wild swine, feral pigs, or feral hogs, are one of the most widespread and successful invasive species around the world. Wild pigs have been linked to extensive and costly agricultural damage and present a serious threat to plant and animal communities due to their rooting behavior and omnivorous diet. We modeled the current distribution of wild pigs in the United States to better understand the physiological and ecological factors that may determine their invasive potential and to guide future study and eradication efforts. Using national-scale wild pig occurrence data reported between 1982 and 2012 by wildlife management professionals, we estimated the probability of wild pig occurrence across the United States using a logistic discrimination function and environmental covariates hypothesized to influence the distribution of the species. Our results suggest the distribution of wild pigs in the U.S. was most strongly limited by cold temperatures and availability of water, and that they were most likely to occur where potential home ranges had higher habitat heterogeneity, providing access to multiple key resources including water, forage, and cover. High probability of occurrence was also associated with frequent high temperatures, up to a high threshold. However, this pattern is driven by pigs’ historic distribution in warm climates of the southern U.S. Further study of pigs’ ability to persist in cold northern climates is needed to better understand whether low temperatures actually limit their distribution. Our model highlights areas at risk of invasion as those with habitat conditions similar to those found in pigs’ current range that are also near current populations. This study provides a macro-scale approach to generalist species distribution modeling that is applicable to other generalist and invasive species.

## Introduction

Globally, invasive species are inflicting increasing amounts of economic and environmental damage on agricultural and ecological systems [1,2]. In the United States (U.S.), the economic cost of invasive species has been estimated to be $120 billion per year [3]. Much of this cost represents losses incurred by agricultural industry. Conflicts between both native and non-native wildlife species and agriculture are increasingly challenging the ability of institutions to mitigate their negative consequences [4,5]. Invasive species are also one of the most serious threats to biodiversity conservation [6] and have been identified as the primary factor threatening approximately 42% of all species of conservation concern in the U.S. [3]. The threats that invasive species pose to both agricultural and ecological systems may continue to increase in future decades along with the increased globalization of commerce [7,8].

Vertebrates are particularly successful invaders. Jeschke and Strayer [9] found that approximately 50% of introduced vertebrate species exchanged between North America and Europe successfully establish, and 50% of those successfully spread. In the U.S., at least 30 species of exotic free-ranging mammals have become established since European colonization [10,11]. These species often become serious pests that negatively impact native species and their environments [11,12]. Large mammals, such as ungulates, are particularly successful invaders due to their intelligence (i.e., large brain sizes), irruptive population dynamics, and declining abundance of predators [13–17].

Wild pigs (*Sus scrofa*), the ungulate species that includes feral and domestic pigs (*S*. *s*. *domesticus*), several subspecies of the wild boar (*S*. *s*. spp.) [18], and hybrids, are one of the world’s most widely distributed mammals [19]. The native range of *S*. *scrofa* is Eurasia and Northern Africa [20], thus wild pigs are an invasive species throughout much of their current geographic range. In North America, wild pigs were first introduced in the 14^th^ and 15^th^ centuries by Spanish explorers in the southern U.S. Wild pigs have recently expanded their North American range to include at least 38 states and three provinces in Canada [21,22]. Wild pig populations continue to increase due to their reproductive capacity, adaptability to novel environments, and intentional or accidental introduction by humans [21,23,24].

The range expansion of wild pigs has resulted in substantial impacts to agricultural production, human food safety, ecosystems, and threatened species in the U.S. There is a well-established association of wild pigs with agricultural and environmental damage, though precise estimates of the economic cost of wild pig damage are limited [21,25]. Wild pigs also present considerable risks to human health through environmental contamination of water and agricultural crops [26] or through direct human exposure to bacterial, viral, or parasitic pathogens [27,28]. The rooting behavior and omnivorous diet of pigs can have dramatic ecosystem-level effects on soil properties as well as plant and animal communities [20,29].

Despite their considerable impacts on agricultural and ecological systems, little is known about environmental factors that most strongly influence the broad-scale distribution and spread of the species in the U.S. In this context, the principal objectives of this study was to evaluate physiological and environmental factors associated with the current distribution of wild swine in the contiguous U.S., and to use these factors to predict where pigs may be most likely to occur (and potentially establish and spread) beyond their current documented U.S. range.

## Methods

### Swine distribution data

The National Feral Swine Mapping System [30], collected and maintained by Southeastern Cooperative Wildlife Disease Study (SCWDS), describes the distribution of wild pigs across the lower forty-eight United States. This spatially explicit dataset, compiled at variable intervals from 1982 to 2004 and annually since 2008, consists of polygons describing the known geographic extent of established wild pig populations that have been present for two or more years and have evidence of reproduction. Data are reported nationally from wildlife professionals in state wildlife resources agencies and the United States Department of Agriculture via manual drawing of polygons using topographical maps to reference areas where pigs have been observed.

**Fig 1:**
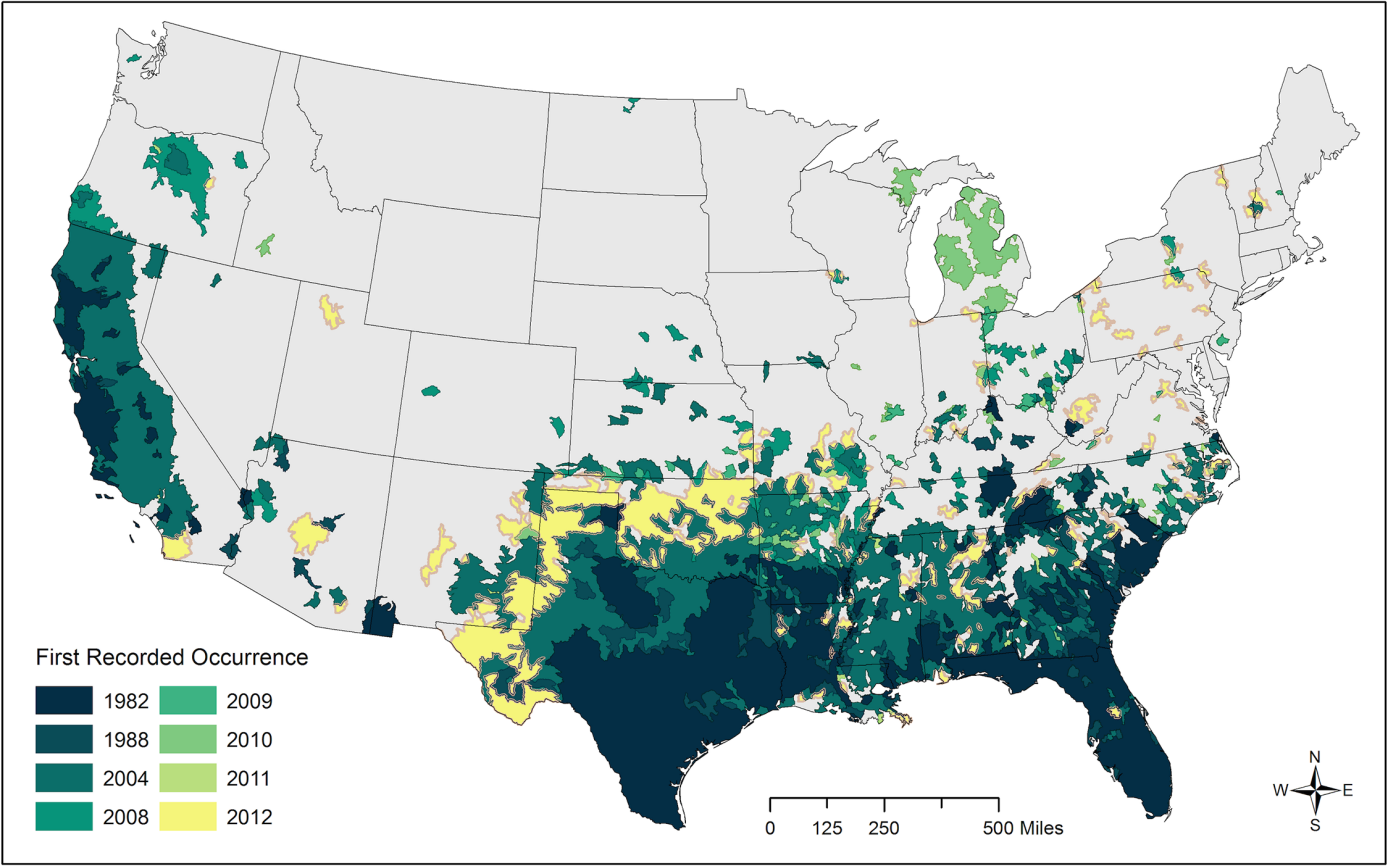
Spread of wild pigs in the contiguous United States. This map illustrates cumulative documented occurrence of wild pigs from 1982 to 2012 based on Southeastern Cooperative Wildlife Disease Study (SCWDS) records aggregated to watersheds (Hydrologic Unit Code 10). Areas occupied by wild pigs in a given year continue to be occupied in later years, with rare exception.

We aggregated the SCWDS data to watersheds described by the United States Geological Survey's (USGS) Hydrologic Unit Codes (HUC) database (HUC10; mean area of 512 ± 255 km^2^) [31]. Aggregation of the original polygon data to discrete sampling units was necessary because drawn polygons varied greatly in size and detail (e.g., virtually all of Texas and most of California were each encompassed by single large polygons), and did not represent consistent, comparable sampling units. We chose watersheds as our sampling unit because they are ecologically relevant landscape-level sampling units for large scale studies [32–34], and have been used to model the occurrence of other species [35,36]. Furthermore, watersheds are expected to represent a more discrete set of biotic and abiotic factors and thus serve as a more ecologically relevant unit for aggregating covariates than an arbitrary rectangular grid. We chose a watershed size (HUC10) that was much larger than the mean home range size estimated for wild pigs in the U.S. (4.92±6.37 km^2^) and thus was expected to be capable of encompassing an entire population of pigs. (S1 Table). To aggregate the SCWDS data to the watershed level (Fig 1), we used two criteria to decide whether or not to assign pig presence to a watershed: (a) the area of a SCWDS pig population (polygon) had to be greater than three times the mean home range size of wild pigs in the U.S. (13 km^2^; 5 mi^2^) or approximately three times the national mean home range size, and (b) the proportion of each watershed occupied by a given population had to be greater than 2.5%. The first criterion ensures that the occupied portion of a watershed is large enough to support multiple wild pig home ranges, and the second criterion ensures that the occupied portion of large watersheds is large enough relative to total size for covariate values to be meaningfully linked to wild pig presence.

In addition to spreading locally through population growth and natural dispersal, wild pigs are occasionally introduced to novel locations by humans for hunting. Because patterns generated by anthropogenic spread are not driven by all of the same ecological factors influencing natural spread, we excluded likely introductions from the distribution data. Based on published estimates of annual dispersal capabilities and the distribution of observed distances between newly occupied watersheds and watersheds occupied in the previous year, we excluded new pig populations that were highly likely to have been introduced by humans from further analysis (4.4% of all records). Note that if a watershed that was deemed to harbor an introduced population continued to be occupied in subsequent years or if adjacent watersheds were later reported as occupied, these occurrences were included in analyses.

### Model Covariates

**Fig 2:**
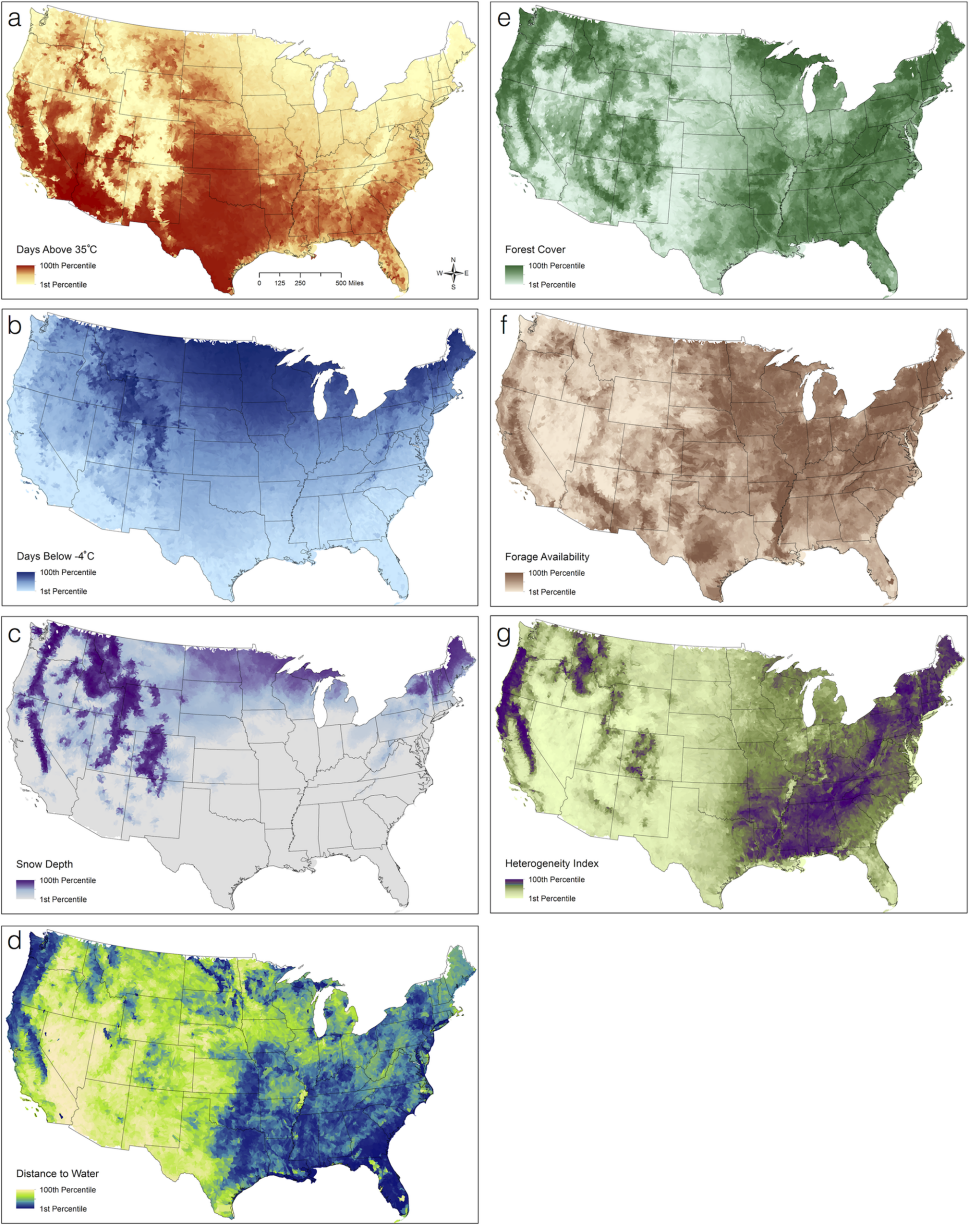
Occurrence model covariates. Mapped covariate layers used to model wild pig occurrence probability across the contiguous United States. All covariate values are depicted using a quantile classification.

We identified physiological constraints and ecological requirements that we hypothesized may influence the observed and potential occurrence of feral swine across the contiguous U.S. We then identified covariates that best represented these factors, which include physiological limits imposed by temperature, access to water, and thermal cover, and ecological requirements for forage and protective cover (Table 1). We used a geographic information system (GIS) to derive spatial data layers for all covariates at the local watershed (HUC10) level across the contiguous U.S. from publicly available national-scale datasets (Fig 2), then standardized all covariates prior to model fitting (data are available: doi:10.5061/dryad.vt46n).

**Table 1:** Covariates used to model wild pig occurrence probability.

Name	Description	Data Source(s)	Spatial Resolution	Temporal Resolution
**Days above 35°C**	Mean number of days above 35°C across weather stations within 250 km of watershed centroid and 30 years of observations	NOAA^a^ weather stations	Point data interpolated to watershed (512 ± 255 km^2^) centroids	30-year mean (1983–2012)
**Days below -4°C**	Mean number of days below -4°C across weather stations within 250 km of watershed centroid and 30 years of observations	NOAA weather stations	Point data interpolated to watershed centroids	30-year mean (1983–2012)
**Snow depth**	10-year mean snow depth on April 1 (estimated annual maximum)	NSIDC SNODAS^b^	30 arcsec	10-year mean (2003–2012)
**Distance to water**	Mean distance to nearest perennial stream or water body perimeter	USGS NHDPlus^c^	1:100,000	1999–2012
**Forest cover**	Percent area with deciduous, evergreen, or mixed forest cover	USGS NLCD^d^ 2006	30 m	2006
**Forage availability**	Percent area classified as hard mast-producing or crop cover	USGS GAP^e^ Land Cover, NASS^f^ CropScape	30 m	GAP: 1999–2001 CropScape: 2012
**Heterogeneity index**	Mean number of key habitat elements (water, cover, forage) available within radius defined by average sounder home range size	NHD Plus, NLCD 2006, GAP Land Cover, NASS CropScape 2012	30 m	Varies; see water, cover, and forage above

Covariates represent estimated physiological temperature limits and ecological requirements for access to water, thermal and protective cover, and forage.

^a^National Oceanic and Atmospheric Administration.

^b^National Snow and Ice Data Center Snow Data Assimilation System.

^c^United Stated Geological Survey National Hydrography Dataset Plus.

^d^United States Geological Survey National Land Cover Dataset.

^e^United States Geological Survey Gap Analysis Program.

^f^National Agricultural Statistics Service.

Pigs are known to have physiological characteristics making them sensitive to both high and low temperatures [37–39]. Porter and Gates [40] (1969) report that pig mortality results from exposure to full sun when ambient temperatures exceed 23°C and exposure to partial sun when ambient temperatures exceed 35°C. The Swine Care Handbook [41] recommends that cooling be provided when temperatures exceed 35°C for domestic pigs of most growth stages and that supplemental heat should be provided to juvenile pigs when temperatures fall below -4°C. We derived the cumulative number of days above 35°C and below -4°C for each watershed in a given year from National Oceanic and Atmospheric Administration (NOAA) weather station data. We identified weather stations within 250 km of each watershed centroid (up to 10 closest stations), then calculated the number of days each station had an observed maximum temperature above 35°C and the number of days with an observed minimum temperature below -4°C. We then adjusted for the difference in elevation between each weather station and the watershed centroid using the average adiabatic lapse rate temperature correction formula [42]:

ΔT=6.49°C/1000m

where ΔT represents a change in temperature of 6.49°C for every 1000 meters of elevation gained or lost between the weather station location and the watershed centroid. We averaged across the selected weather stations and over 30 years of observations, or all years in a 30 year time period for which data were available.

Wild pig survival and reproductive success at low temperatures in natural environments is expected to be influenced by snow presence and depth [39,43,44]. Mean snow depth was estimated from the Snow Data Assimilation System (SNODAS), which integrates snow data from satellite platforms, airborne platforms, ground stations, and models to estimate snow cover and depth [45]. Using estimates from April 1, which is assumed by most resource managers to be the date closest to maximum snow accumulation in temperate latitudes of the northern hemisphere [45], we calculated the average maximum snow depth over 10 years, then averaged within each watershed.

Wild pigs thermo-regulate by accessing shade and water resources [46,47], and restricted access to water is known to cause increased piglet mortality [48]. Mean distance to water was derived from the National Hydrography Dataset Plus (NHDPlus) [49]. First, streams with very low average annual flow (< 3 cubic feet per second) were removed to exclude ephemeral water sources. Distances from remaining stream features and water body perimeters were then measured and summarized by watershed to yield the average distance to the nearest water source from any grid cell within each watershed. Forest canopies also offer important thermal as well as protective cover for wild pigs [44,46,50]. Availability of forest cover was derived from the 2006 National Land Cover Dataset by calculating the percent area of each watershed classified as deciduous forest, coniferous forest, mixed forest, or woody wetlands cover.

Wild pigs are known to be a highly adaptable generalist species in terms of their dietary range [51]. We identified two major forage classes typically available to wild swine at the national scale, crops [37,39] and hard mast (i.e., acorns and other nuts) [37,44,52]. Crop cover was derived from the USDA National Agricultural Statistics Service Cropland Data Layer (2012). All crop types were included as potential forage resources given that wild pigs are known to consume a diverse array of crops, as well as insects and other food sources associated with crops [51]. Mast-producing cover was derived from the USGS Gap Analysis Program (GAP) National Land Cover dataset v2 (2011). We screened cover class names and descriptions to identify those dominated by or containing a significant presence of hard mast-producing tree or shrub species; including oak (*Quercus spp*.), hickory (*Carya spp*.), chestnut (*Castanea spp*.), walnut (*Juglans spp*.), beech (*Fagus spp*.), birch (*Betula spp*.), maple (*Acer spp*.), elm (*Ulmus spp*.), and ash (*Fraxinus spp*.). We calculated the proportion of area within each watershed classified as crop or mast-producing cover as an index of forage availability.

Finally, we derived an index of habitat heterogeneity representing the availability of all three of the resources that meet wild pigs’ key ecological and physiological requirements–water, cover, and forage–within the average group home range area as estimated in previous studies (9 km^2^ based on 95–100% utilization distributions for mixed groups or sounder groups; S1 Table). By applying a moving window approach to raster maps of each habitat component, we calculated the number of components present within the area of an average sounder range centered at each focal cell. We then averaged these counts across each watershed to generate a continuous index (0–3) of habitat heterogeneity.

### Model fitting

Within an information-theoretic framework [53,54], we used logistic regression and multi-model inference [54–56] to estimate a logistic discrimination function [57,58] representing the relative probability of occurrence of wild pigs. The function discriminates between watersheds where a species is present and random ‘background’ watersheds based on the distributions of covariates associated with each. This approach is similar to fitting a resource selection function (RSF) [59], but differs in that presence and background watersheds are sampled independently, allowing watersheds occurring in the presence sample to also occur in the background sample. The logistic discrimination function avoids the problematic assumption that background watersheds represent absences or ‘pseudo-absences’, but rather reflects the probability of species occurrence given the distribution of habitat covariates at presence watersheds, relative to background watersheds. While other more recent methods of estimating occurrence probability from presence-only data were considered (e.g., MaxEnt [60], MaxLike [61], scaled binomial loss (SBL) [62], presence-background learning algorithm (PBL) [63]), each has been shown to fail to estimate a quantity proportional to absolute probability of occurrence as estimated by more complete presence-absence data, either in general (MaxEnt [61,64–66]) or when required parametric assumptions regarding species prevalence (MaxLike [62,67]) or empirical estimates of prevalence (SBL, PBL) were not accurate. We instead chose a simpler approach with transparent interpretation that we expected to be more robust for this application.

The presence sample included all watersheds in which wild pigs were reported throughout the sampling period (1982–2012), except those identified as likely recent human introductions (N = 4459). We sampled background locations from all watersheds in the contiguous United States, including those with recorded presences. We selected a background sample size that was twice that of the presence sample and approximately half of all contiguous U.S. watersheds. Although selection of the background sample size was arbitrary and a larger sample could have been selected, this approach avoided both inflating the degrees of freedom in our model and excessive overlap of presence and background samples, though the logistic discrimination model is robust to sample overlap [57].

Our global model included the entire suite of covariate (*j*) linear terms, along with a quadratic term for number of days above 35°C because we hypothesized increased probability of occurrence in warmer climates up to a threshold beyond which additional hot days would be detrimental to pigs. We tested for collinearity by calculating pairwise Pearson correlations and variance inflation factors, but no terms exceeded cutoff values of 0.7 or 10.0, respectively [68,69], and thus no exclusion of terms from the model was necessary.

We used all-subsets model averaging and multi-model inference to arrive at a final predictive logistic discrimination function. Rather than base inferences and prediction on a single, selected ‘best’ model from an a priori set of models, more robust inference can be based on the entire set of models considered [54,55]. Model averaging across all model subsets produces parameter and error estimates that are not conditional on any one model but are instead informed by the entire model set [54,55,70]. This is particularly advantageous when several models have similar weights of evidence, or probability of being the ‘best’ model [56]. Averaging over all possible subsets of a global model is recommended over selection of candidate model sets when the aim is to produce a model averaged predictive model, provided there is strong support for inclusion of each covariate in the global model to avoid a ‘fishing expedition’ [70]. The superiority of model-averaged inferences compared to a traditional ‘best’ model selection strategy has been demonstrated repeatedly (e.g., [55,71,72]).

We used the ‘dredge’ and ‘model.avg’ functions in the MuMIn package [73] for R [74] to fit all additive subsets of the global model and compute model-averaged regression coefficients, unconditional standard errors (SEs), cumulative AIC weights of evidence as a measure of variable importance [54–56], and 95% confidence intervals [54,55]. We used a shrinkage estimation approach to produce unconditional model averaged parameter estimates, in which covariates that did not appear in a particular model subset were assigned coefficients of zero to avoid biasing coefficient estimates away from zero [54,75]. Our interpretation of the explanatory power of the regression coefficients in our model was guided by three measures: 1) the weights of evidence, ranging from 0 to 1.0, where higher weights indicated greater relative importance; 2) the 95% confidence interval for each regression coefficient; and 3) effect sizes indicated by each regression coefficient.

### Model validation

Standard model validation metrics test discrimination between presence and absence locations and are thus not appropriate for testing the predictive performance of a model designed to discriminate between presence and background locations [76]. We instead used the “RSF plot index”, a variation of k-fold cross-validation designed for presence-only data, to assess proportionality of the relative probability of occurrence predicted by the model and the observed frequency of occurrence [76]. Based on Huberty’s rule [77], we first randomly divided the wild pig data among four cross-validation folds. We used each possible set of three folds to fit a predictive model, again employing multi-model averaging, which we then used to predict the fourth withheld fold. Results of 100 iterations of this process, each with a new random allocation of data across four cross-validation folds, were averaged to avoid dependency of validation results on a single random allocation of data across folds.

We binned predicted values from our cross validation results, then calculated a Pearson correlation between those values and the proportion of watersheds within each bin for which the species was recorded as present. Because validation results can be sensitive to binning method [76], we applied and compared both equal interval and quantile binning methods. Lastly, we assessed the performance of our final model using the Pearson correlation rather than the Spearman rank correlation as in Boyce et al. [76] because the former provides a more rigorous measure of the linear agreement between predicted probability of occurrence and observed frequency of occurrence.

## Results

Based on the final inferential model (Table 2), the distribution of wild pigs in the contiguous U.S. was most strongly limited by frequent cold temperatures and the availability of water and is most strongly associated with frequent high temperatures and high habitat heterogeneity within a home range. Covariates representing each of these four factors had AICc weights of evidence of 1.0, indicating high importance.

**Table 2:** Summary of the final inferential model.

Variable	Estimate	SE	95% CI		AIC weight
Days Above 35°C	0.638	0.044	0.5518	0.7242	1.00
(Days Above 35°C)^2^	-0.120	0.012	-0.1435	-0.0965	1.00
Days Below -4°C	-2.749	0.106	-2.9568	-2.5412	1.00
Snow Depth	-0.006	0.020	-0.0452	0.0332	0.31
Distance from Water	-0.529	0.053	-0.6329	-0.4251	1.00
Forest Cover	-0.035	0.039	-0.1114	0.0414	0.60
Forage Availability	0.009	0.020	-0.0302	0.0482	0.36
Heterogeneity	0.197	0.047	0.1049	0.2891	1.00

Model-averaged parameter estimates, unconditional standard errors (SE), 95% confidence intervals (CIs), and cumulative Akaike’s Information Criterion weights for all covariates used to model the relative probability of occurrence within each watershed across the contiguous U.S.

Covariates representing frequency of cold temperatures and distance from water had significant negative model coefficients, indicating a decrease in the relative probability of wild pig occurrence with increasing average number of days below -4°C (standardized 95% CI: -2.9563 –-2.5413) and with increasing distance from water (standardized 95% CI: -0.6329 –-0.4259). In terms of effect sizes, for each additional day with observed minimum temperature below -4°C per year within a watershed, there was an estimated 15% decrease in the odds of wild pig occurrence, and for each 1 km increase in the average distance to water from a given location in a watershed, there was an estimated 27% decrease in the odds of wild pig occurrence.

Habitat heterogeneity had a significant positive model coefficient, indicating an increase in the relative probability of wild pig occurrence with increasing habitat heterogeneity (95% CI: 0.1045–0.2887). The availability of an additional heterogeneity component (i.e., water, forage, cover) within an average home range area increased the odds of wild pig occurrence by 32%.

Our final model contained a significant positive linear coefficient (95% CI: 0.5523–0.7229) and a significant negative quadratic coefficient (95% CI: -0.1431 –-0.0966) for average number of days above 35°C. Together, these coefficients indicated that the odds of wild pig occurrence increased with the number of days with maximum temperature above 35°C up to an asymptote of approximately 59 days per year, beyond which additional days with maximum temperature above 35°C reduced the odds of occurrence. This threshold occurs well above the observed mean of 14 days with maximum temperature above 35°C across the contiguous U.S. (SD = 22 days).

The linear terms of forest cover, forage availability, and snow depth had AICc weights of evidence of 0.6, 0.36, and 0.31, respectively, and did not have significant effects on the relative probability of wild pig occurrence based on 95% confidence intervals on estimated coefficients.

The top-ranked model had an AICc weight of 27% (given the candidate set) and was 4054 AICc units better (i.e., lower) than the null model, suggesting that the selected suite of covariates approximated the data well.

**Fig 3:**
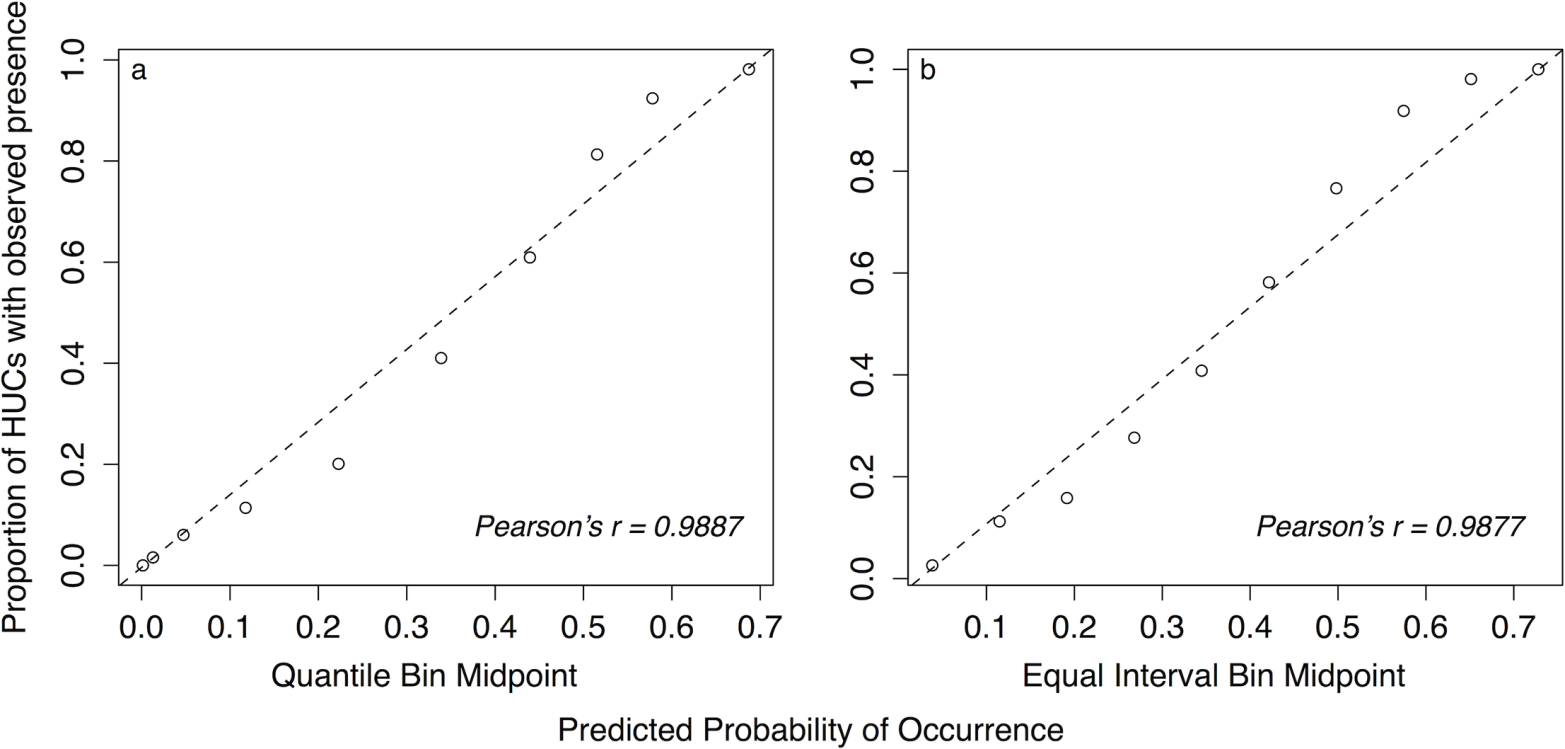
Cross validation results. Estimation of the predictive capacity of the wild pig occurrence model based on RSF plots using a) quantile and b) equal interval binning methods.

Cross-validation based on the RSF plot index indicated that the final model had strong predictive capacity (Fig 3). The quantile binning method (equal numbers of watersheds in each bin) produced a Pearson correlation of 0.989 between midpoints of predicted probability of occurrence values and observed proportions of occupied watersheds in each bin. Similarly, the equal interval binning method produced a Pearson correlation of 0.988, indicating low sensitivity of the cross-validation results to binning method.

**Fig 4:**
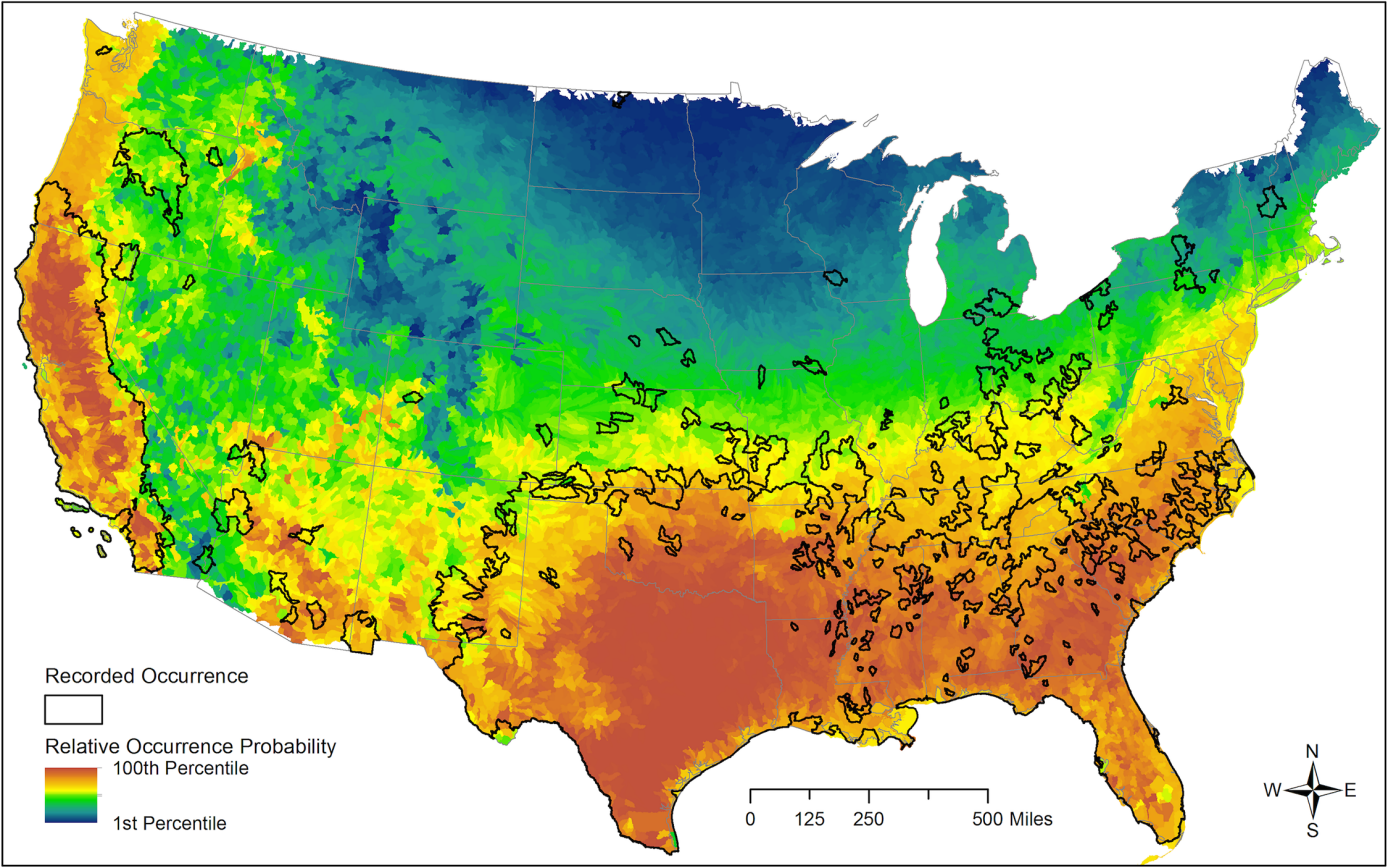
Predicted wild pig occurrence. Predictive map of relative wild pig occurrence probability based on a logistic discrimination function relating Southeastern Cooperative Wildlife Disease Study (SCWDS) records collected from 1982 to 2012 with covariates representing ecological and physiological requirements, with actual reported distribution overlaid.

We used the exponential form of our final model to predict the relative probability of wild pig occurrence across the contiguous United States (Fig 4). As expected, high occurrence probabilities were predicted in the South, generally aligning well with known wild pig occurrence, while low probability of occurrence was predicted in cold regions (e.g., Rocky Mountains, Northern Great Plains) and in arid regions (e.g., desert regions of Nevada and inland southern California).

## Discussion

Validation results indicated that our model performed well in predicting high occurrence of wild pigs over much of the species’ current U.S. range, while also identifying additional areas that may be capable of supporting populations of wild pigs that are, apparently, unoccupied, which is perhaps of greatest interest and utility (Fig 4). These areas, such as the Pacific Northwest, mid-Atlantic region, and Southwest, have similar climatic and habitat conditions as areas within the species’ current U.S. range and are also near known populations. The mechanisms for wild pig dispersal in the United States are largely unknown, but human-mediated dispersal may be a key cause of population spread in recent decades [11]. Recent analyses indicated that genetic sources of wild pigs throughout their United States range include genes from historic wild pig populations in the Southeastern United States, as well as introduction of new sources [78]. The full range of genetic sources is not yet characterized but could include escape or intentional release of domestic pigs or farmed wild boar. Although our model does not account for dispersal, the high-risk areas we identified represent key geographic areas warranting surveillance to identify newly established wild pig populations, whether through translocations from existing populations or propagules from agricultural operations.

Temperature is a key limiting factor for many species, especially at higher latitudes [79], and is often used to predict species distributions [80]. There is a well-established morbidity response of pigs to both high and low temperatures in captive conditions. A review of 54 wild boar population density estimates from Eurasia found that low winter temperatures were associated with smaller populations [43]. Our results suggest that this physiological limitation may also be present for wild pigs in the contiguous U.S. However, the current data available for the known distribution of wild pigs is confounded with higher temperature regions where wild pigs have been present longest in the U.S., which was influenced by the original introductions in the Southeastern states. Hence, we suggest there is a need to collect more data in Northern regions of the U.S. and in Canada where wild pigs may be underreported due to historic absence and/or lower density.

Wild pigs are in the same taxonomic order (Cetartiodactyla) as other large mammalian herbivores and, despite their omnivorous food habits, vegetation still comprises most of their diet [51]. Fine-scale telemetry studies have found wild boar and feral pigs using a range of natural and anthropogenic habitats to access either food or cover [39,81,82], and we suspected pigs might show a similar positive relationship between presence and habitat heterogeneity as other large mammalian herbivores [83–86]. The response by ungulates to heterogeneity often differs based on the spatial scale of at which heterogeneity is measured [87]. Most previous ungulate-heterogeneity studies evaluated responses from local (i.e., patch or home-range) to landscape scales and, interestingly, often found ungulates showed the strongest positive response to heterogeneity at the broader landscape scale [83,87]. By measuring heterogeneity at the scale of a home range and then summarizing these measurements across watersheds, our study helps extend ungulate-heterogeneity studies beyond landscape scales to evaluate whether heterogeneity influences their distribution at a near-continental scale.

The individual components of our habitat heterogeneity index had different influences on the occurrence of wild pigs (Table 2). For example, the probability of pig occurrence decreased with increasing average distance to water, as predicted based on pigs’ physiological dependence on behavioral thermoregulation. In contrast, forest cover and forage availability showed little association with the current distribution of wild pigs in the U.S. These results were somewhat surprising because food and cover are usually influential covariates in species distribution models [80]. We suspect their minimal influence here results from the generalist food and habitat affinities of wild pigs. However, the collective presence of food, cover, and water summarized as a metric of habitat heterogeneity at a landscape scale had a strong positive relationship with the distribution of wild pigs in the U.S. While this result may be strongly influenced by water availability, it still provides evidence that access to three critical resources of food, cover, and water is a critical aspect of landscapes currently supporting pig populations in the U.S.

Although wild pigs present a growing problem as an invasive species in North America, they also represent many of the characteristics that make generalist species successful invaders. Notably, their highly plastic diet [51] and adaptability to novel habitats [82] present challenges to identifying the drivers of distribution and spread of this generalist species. By taking a macro-scale (continental) view of the wild pig distribution in the U.S., our modeling approach confirmed that wild pigs are extreme generalists. We not only quantified a wide range of habitats in which pigs had a high relative probability of occurrence, but also identified regions that are within the environmental conditions known to support pig populations in the U.S. but are apparently unoccupied. Such insights would not be revealed with local-scale data. We recommend that, whenever feasible, similar macro-scale approaches to generalist species distribution modeling are pursued to capture the extent of conditions that support a species population and generate hypotheses about species limitations or invasion potential that can be tested in combination with finer-scale research.

Our model identified some areas with long established populations that had only moderate predicted occurrence probabilities (e.g., portions of Florida). This is largely due to the strong influence of temperature on the model and the overall positive relationship between high temperatures and occurrence driven by pigs’ historical distribution in the U.S. As a result, coastal areas with mild temperatures relative to nearby inland areas are predicted to be less likely to harbor pigs despite otherwise favorable conditions and historic pig presence. Furthermore, occurrence models are not designed to identify a threshold probability below which pigs are not expected to occur. Most areas with moderate occurrence probabilities are expected to be highly suitable for pigs given their generalist nature and capacity for adaptation to a variety of conditions. Even areas with very low occurrence probabilities (e.g., Northern states) may be capable of supporting pig populations, though perhaps with lower establishment success or at lower densities than in more favorable areas [22].

## Conclusions

In this paper, we identified the areas currently occupied by wild pigs in the contiguous United States and, more importantly, predicted those areas that would most likely support pigs if colonized in the future. Areas predicted to be highly suitable for pigs that are not currently occupied but that are near wild pigs’ current range may be particularly at risk of invasion. We suggest that this information, particularly when coupled with spatial patterns of agricultural production, biodiversity indices, or the distribution of species and habitats that may be sensitive to the impacts of pigs, can help guide prioritization of wild pig management practices so as to minimize the impacts of spreading pig populations on agricultural and ecological systems.

Although large portions of the contiguous U.S. are predicted to have very low probability of wild pig occurrence, recent studies have shown that wild pigs can occur in environments previously thought to be inhospitable, for example in Saskatchewan, Canada [22]. Thus, future work is needed to improve our understanding of the drivers of wild pig occurrence throughout North America, particularly the extent to which cold temperatures actually limit wild pig colonization and establishment. A critical first step in this process would be to obtain new and improved occurrence data for wild pigs in the northern U.S. and Canada. Wild pigs are expected to occur at lower densities in these regions, which may make detection of populations difficult. However, development of cost-effective and widely distributable surveys to state wildlife managers is one approach that should be considered. In addition, more field data is needed along a latitudinal gradient to understand how the determinants of occurrence, space-use, and vital rates vary at this continental scale. This may result in a set of regional occurrence models with potentially different driving covariates. Future work must also seek to estimate wild pig population size, and how it varies, across the contiguous U.S., which will result in improved updates to our national-level species distribution model and help managers identify locations requiring active management of wild pig populations.

Finally, one of the largest challenges limiting the understanding and management of recent wild pig range expansion concerns the mechanisms of spread. Our work, in combination with distribution modeling in Canada [22], suggests that much of North America has suitable conditions for wild pigs, including portions that are currently unoccupied. We suspect that the absence of wild pigs in suitable, yet apparently unoccupied habitat results from lack of introduction (i.e., the propagule pressure hypothesis) [88], as opposed to limiting environmental or habitat factors that our distribution model failed to capture. Although dispersal capacity and long-term movement data for wild pigs is lacking in North America, we also suspect that social factors such as value of wild pigs as a recreational hunting resource or as farmed species are at least as important as natural dispersal in driving the current distribution of wild pigs. As such, we recommend that future research investigating the distribution and invasiveness of wild pigs should include social factors that may drive value and motivation for human translocation of wild pigs (*what drives wild pig propagule pressure*?), in addition to biological factors (*what biotic and abiotic factors limit wild pigs populations*?) to address competing hypotheses and generate effective management solutions.

## Supporting Information

S1 Table Review of wild pig home range size estimates. This supporting table compiles wild pig, feral hog, and wild boar home range size estimates available in published literature. Area estimates are given along with information regarding location, number of individuals, estimation method used, and other supporting details.
(XLSX)
